# MSP-RON Signaling Is Activated in the Transition From Pancreatic Intraepithelial Neoplasia (PanIN) to Pancreatic Ductal Adenocarcinoma (PDAC)

**DOI:** 10.3389/fphys.2019.00147

**Published:** 2019-02-26

**Authors:** Ce Li, Susan Morvaridi, Gloria Lam, Chintan Chheda, Yoshiko Kamata, Makoto Katsumata, Mouad Edderkaoui, Xiaopu Yuan, Nicholas Nissen, Stephen J. Pandol, Qiang Wang

**Affiliations:** ^1^Department of Medical Oncology, The First Affiliated Hospital of China Medical University, Shenyang, China; ^2^Department of Medicine, Cedars-Sinai Medical Center, Los Angeles, CA, United States; ^3^Department of Biomedical Sciences, Cedars-Sinai Medical Center, Los Angeles, CA, United States; ^4^Department of Pathology, Cedars-Sinai Medical Center, Los Angeles, CA, United States; ^5^Comprehensive Transplant Center, Cedars-Sinai Medical Center, Los Angeles, CA, United States

**Keywords:** MSP/MST1, RON/MST1R, matriptase, pancreas, stellate cell, pancreatic ductal adenocarcinoma, metastasis, pancreatic intraepithelial neoplasia

## Abstract

Pancreatic ductal adenocarcinoma (PDAC) is among the deadliest epithelial malignancies and remains difficult to treat. Pancreatic intraepithelial neoplasias (PanINs) represent the majority of the pre-cancer lesions in the pancreas. The PDAC microenvironment consists of activated pancreatic stellate cells (PSCs) and immune cells, which are thought to contribute to neoplastic transformation. However, the signaling events involved in driving the transition from the neoplastic precursor to the more advanced and aggressive forms in the pancreas are not well understood. Recepteur d’Origine Nantais (RON) is a c-MET family receptor tyrosine kinase that is implicated in playing a role in cell proliferation, migration and other aspects of tumorigenesis. Macrophage stimulating protein (MSP) is the ligand for RON and becomes activated upon proteolytic cleavage by matriptase (also known as ST14), a type II transmembrane serine protease. In the current study, by immunohistochemistry (IHC) analysis of human pancreatic tissues, we found that the expression levels MSP and matriptase are drastically increased during the transition from the preneoplastic PanIN stages to the more advanced and aggressive PDAC. Moreover, RON is highly expressed in both PDAC and in cancer-associated stellate cells. In contrast, MSP, RON, and matriptase are expressed at low levels, if any, in normal pancreas. Our study underscores an emerging role of MSP-RON autocrine and paracrine signaling events in driving malignant progression in the pancreas.

## Introduction

Pancreatic cancer has extremely poor prognosis and is the fourth leading cause of cancer-related death (Hidalgo, 2010; Jemal et al., 2011; Siegel et al., 2013). Pancreatic ductal adenocarcinoma (PDAC) comprises more than 85% of all pancreatic cancer and has an overall 5-year survival rate of less than 5% (Hidalgo, 2010). A major challenge in the clinics is the lack of effective methods for early detection and treatment. Three types of “preneoplastic” lesions have been characterized as potential precursors of PDAC, including pancreatic intraepithelial neoplasias (PanINs), intraductal papillary mucinous neoplasms (IPMNs), and mucinous cystic neoplasms (MCN) (Hruban et al., 2000; Maitra et al., 2005). In particular, PanINs represent the majority of early neoplastic lesions and are characterized by three morphologically defined stages, namely PanIN1, 2, and 3 (Hruban et al., 2000; Maitra et al., 2005). However, the signaling events involved in promoting the transition from the preneoplastic lesion to the more advanced and aggressive forms are still not fully understood.

Recepteur d’origine nantais (RON), also known as macrophage stimulating 1-receptor or MST1R) is a c-MET family receptor tyrosine kinase (Park et al., 1987; Ronsin et al., 1993). Ligand-dependent or independent activation of RON leads to cell proliferation, migration, and matrix invasion (Lu et al., 2007; Wagh et al., 2008). Aberrant activation of RON has been linked to various forms of human cancers. For example, overexpression of RON is found in the majority of primary human colorectal adenocarcinoma and colon cancer cell lines (Chen et al., 2000; Zhou et al., 2003). In addition, elevation of RON expression has also been found in bladder, head and neck squamous cell carcinomas, breast and ovarian cancers (Maggiora et al., 2003; Lin et al., 2004; Cheng et al., 2005; Lee et al., 2005; Welm et al., 2007). The ligand for RON, known as the macrophage-stimulating protein (MSP) or the hepatocyte growth factor-like protein (HGFL), is a member of the plasminogen-prothrombin family proteins (Wang et al., 1994; Camp et al., 2005; Yao et al., 2013). MSP is expressed as an inactive precursor and becomes activated upon proteolytic cleavage by type II membrane serine proteases, such as matriptase (also known as ST-14) (Bhatt et al., 2007).

Here, we show that elements of the MSP-RON signaling pathway are upregulated in pancreatic cancer cells as well as in cancer-associated pancreatic stellate cells (PSCs). Our results support the notion that activation of MSP-RON signaling represents a hallmark event in progression of PDAC.

## Results

### MSP Is Upregulated in Human PDAC

**FIGURE 1:**
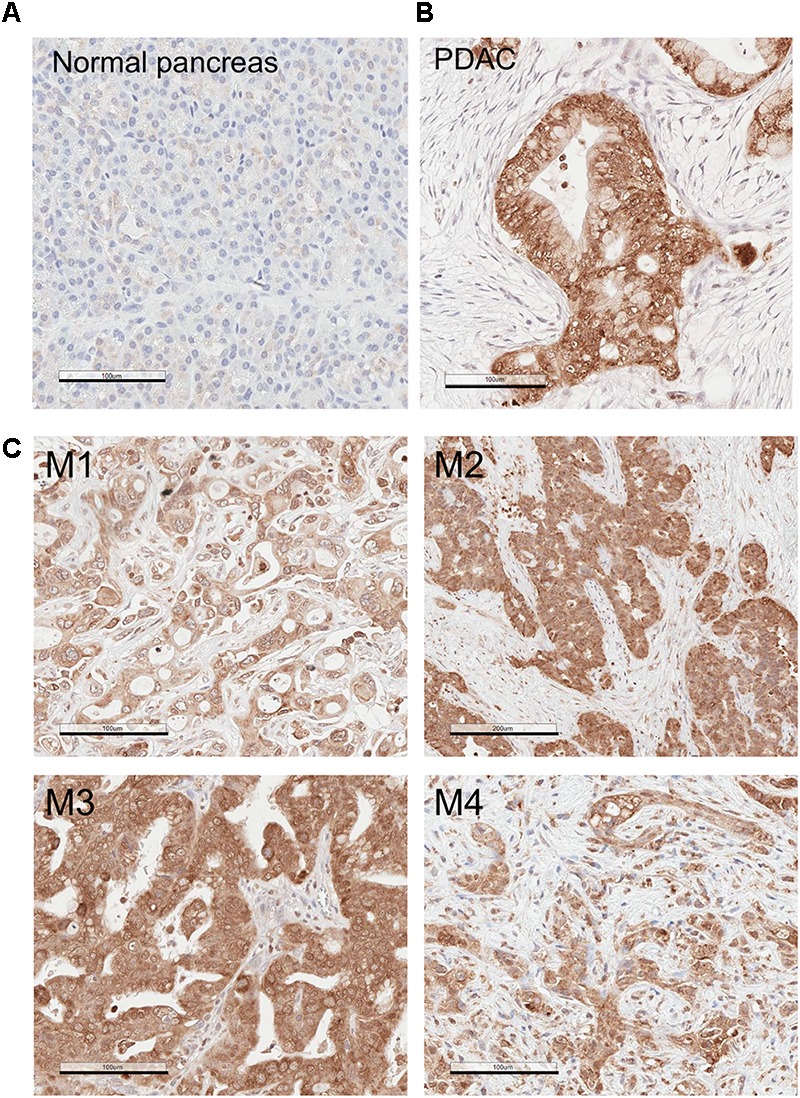
MSP expression is upregulated in Pancreatic ductal adenocarcinoma (PDAC) primary tumors and liver metastasis. Immunohistochemistry (IHC) analysis of human tissues using anti-MSP antibody. **(A)** Normal pancreas; **(B)** PDAC; **(C)** Pancreatic cancer metastasis to the liver. Magnification: 20×; Scale bar: 100 μm.

We examined the expression patterns of MSP in normal human pancreatic tissues and in PDAC by immunohistochemistry (IHC). Our results show that, while MSP expression is minimal in normal pancreas, it is significantly upregulated in the cancer cells of all 12 PDAC specimens that we analyzed (Figure 1A,B). In addition, high levels of MSP can be detected in the pancreatic cancer cells disseminated to the liver in all four samples that we were able to obtain (Figure 1C). We also performed IHC staining on a tissue microarray (TMA) that includes 38 PDAC samples and found that high levels of MSP can be detected in 79% (30 of 38) of the specimens (Table 1).

**Table 1:** Macrophage-stimulating protein (MSP) levels in tissue micro array (TMA) of Pancreatic intraepithelial neoplasias (PanIN), and Pancreatic ductal adenocarcinoma (PDAC).

Tissue type	MSP high	MSP low	*P*-value
PanIN	2	12	
PDAC	30	8	0.0002


*The number of each tissue type with high or low levels of MSP is shown.*

**FIGURE 2:**
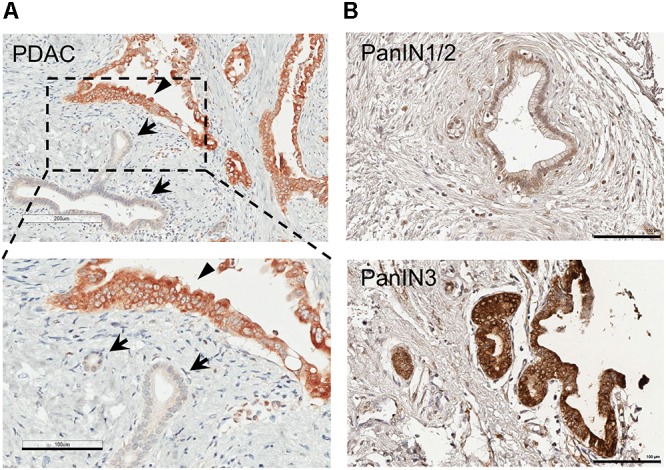
MSP expression in human pancreatic cancer tissues and tissue micro array (TMA). IHC staining of human pancreatic tissues using anti-MSP antibody. **(A)** PDAC. Note that MSP can be detected in cancer cells (arrowheads) but not in normal or un transformed ductal epithelial cells (arrows). **(B)** Representative images of TMA with PanIN1/2 or 3. Magnification: 20×; Scale bar: 100 μm.

We noted that the epithelial cells with morphology of normal or non-transformed ductal cells consistently express little MSP (Figure 2). To determine whether MSP is selectively upregulated during the transition from PanIN to PDAC, we analyzed a TMA that includes fourteen PanIN samples. Interestingly, we found that only 14% (2 of 14) of the PanIN samples were stained MSP positive. These include 0 of 11 specimens that were characterized as PanIN 1/2, and 2 of 3 as PanIN 3 (Figure 2 and Table 1). Although we were not able to obtain sufficient number of tissues with characteristics of PanIN 3, it appears that upregulation of MSP occurs at either the transition to PanIN3 or PDAC, but not in PanIN 1/2.

### RON Levels Are Increased in Pancreatic Cancer Cells and in PDAC-Associated Stellate Cells

**FIGURE 3:**
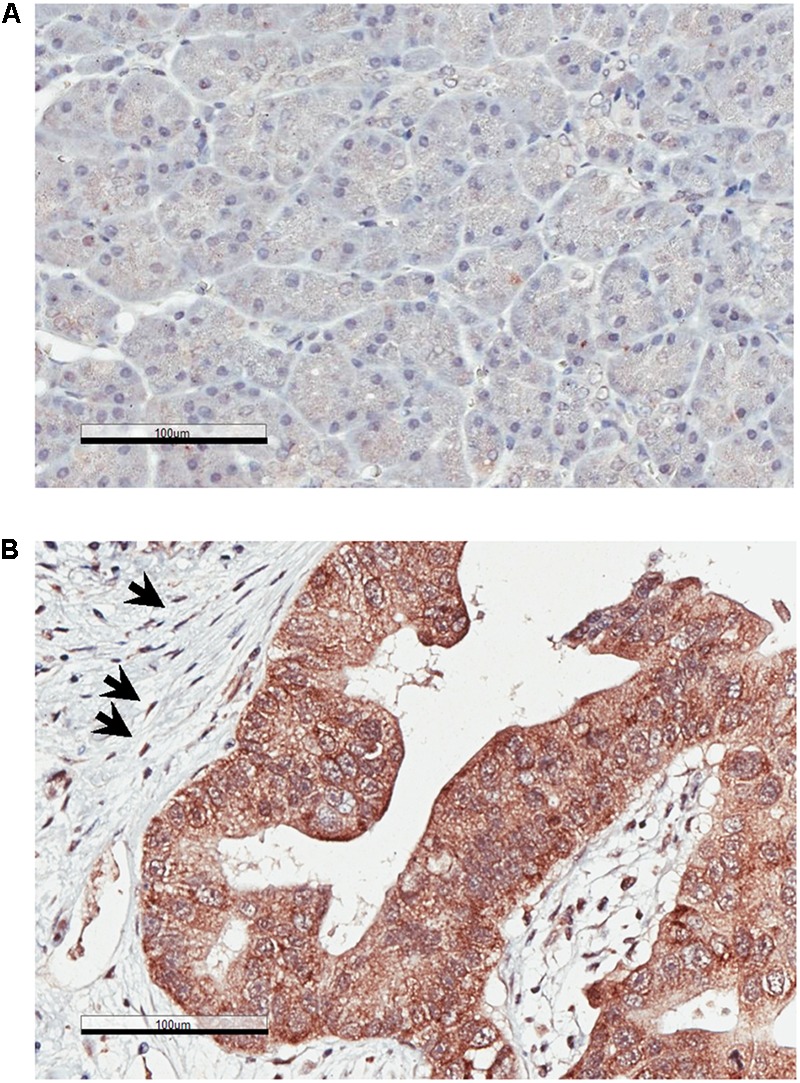
Recepteur d’Origine Nantais (RON) expression is increased in PDAC. IHC analysis of human pancreatic specimens using anti-RON antibody. **(A)** Normal pancreas; **(B)** PDAC. The arrows indicate RON-positive stellate cells. Magnification: 20×; Scale bar: 100 μm.

Our IHC staining show that RON expression levels are significantly higher in PDAC, when compared to normal pancreas (Figure 3). Of note, we found that some stromal cells in PDAC were stained positive for RON (Figure 3). These cells show morphology of stellate cells (Figure 3B; indicated by the arrows). These results indicate that RON is present in both cancer cells and in the PDAC-associated stellate cells.

### Upregulation of Matriptase in PDAC

**FIGURE 4:**
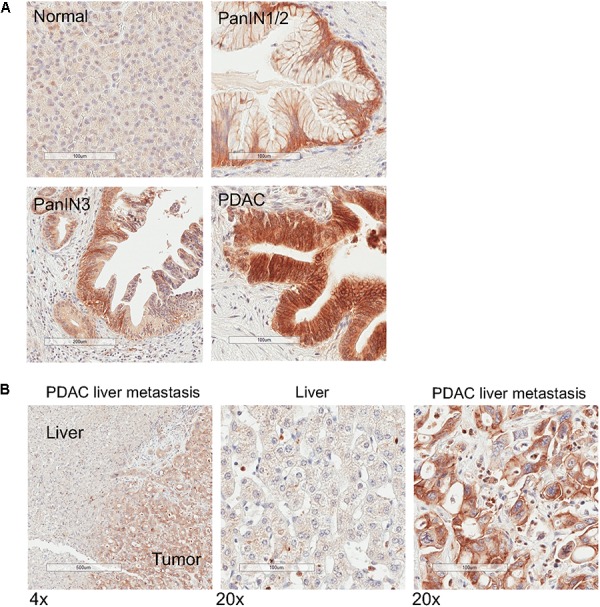
Matriptase expression is upregulated in PanIN, primary PDAC and liver metastasis. IHC analysis of normal human pancreatic tissues using anti-matriptase antibody. Representative staining images are shown. **(A)** Normal pancreas, PanIN1/2, PanIN 3, and PDAC; **(B)** Specimen of pancreatic cancer liver metastasis. The areas of the tumor and the adjacent tumor are indicated. Magnification: 4× (left panel) or 20×; Scale bar: 500 (left panel) or 100 μm.

Because matriptase has been shown to be involved in the processing and maturation of the active form of MSP (Bhatt et al., 2007), we evaluated its expression levels in 12 specimens of normal pancreas and PDAC tissues, respectively. Similar to the expression patterns of MSP, matriptase levels are very low or barely detectable in all normal pancreatic tissues, but can be detected at high levels, notably on the plasma membrane, in the epithelial cells of all PDAC samples that we tested (Figure 4A). Moreover, high levels of matriptase can be detected in the pancreatic cancer cells of all four samples of PDAC liver metastasis that we examined, but not in the hepatic parenchymal cells (Figure 4B). Using a limited number of specimens that show PanIN characteristics, we found that matriptase can be detected in the preneoplastic lesions (Figure 4A; 6 of 7 PanIN1/2; 2 of 2 PanIN3), which indicates that upregulation of matriptase probably occurs earlier than that of MSP.

### MSP Levels Are Elevated in KRAS^G12D^ Mutant-Mediated Pancreatic Intraepithelial Neoplastic Lesion and a Mouse Model of PDAC

**FIGURE 5:**
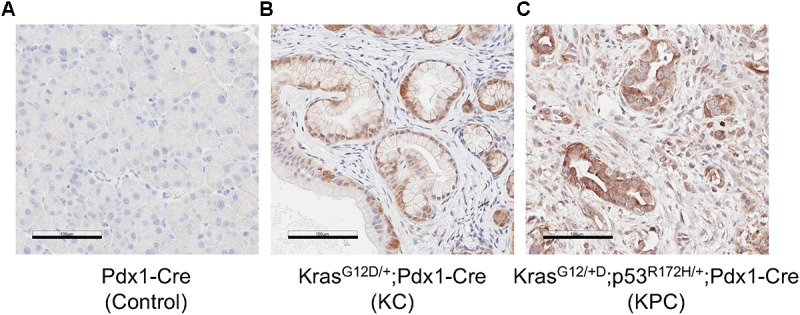
MSP expression in mouse pancreatic tissues. IHC analysis of mouse pancreatic tissues using anti-MSP antibody. **(A)** control (Pdx1-Cre); **(B)** KC mice (LSL-KRAS^G12D/+^; Pdx1-Cre); or **(C)** KPC mice (LSL-KRAS^G12D/+^; LSL-p53^R172H/+^; Pdx1-Cre). Magnification: 20×; Scale bar: 100 μm.

We also examined MSP expression patterns in a transgenic mouse model of pancreatic neoplasm. The KRAS gene is mutated in more than 90% of pancreatic cancer patients (Almoguera et al., 1988; Ryan et al., 2014). Mutations of KRAS, such as G12D, can lead to abnormal activation of KRAS and are thought to be a driver of early neoplastic lesions (Scheffzek et al., 1997). In the Pdx1-Cre/LSL-KRAS^G12D/+^ (KC) transgenic mice, the KRAS^G12D^ mutant is expressed in pancreatic epithelial cells by virtue of the Pdx1 promoter-mediated expression of Cre recombinase and promotes development of PanIN (Hingorani et al., 2003). In addition, co-expression of the p53^R172H^ and KRAS^G12D^ mutants in the pancreas, as in the KPC mice, leads to PDAC (Hingorani et al., 2005). Our IHC studies indicate that, in the control Pdx1-Cre mice, cells in the exocrine pancreas express little, if any, MSP (Figure 5). In contrast, High levels of MSP can be detected in some of cells exhibiting PanIN morphology in the pancreas of the KC mice, and in all PDAC cells in the KPC mice (Figure 5). However, because our antibodies react poorly with mouse matriptase or RON, we were not able to examine these proteins in the KC or KPC mice.

## Discussion

In this study, we show that MSP and the protease responsible for its activation, namely matriptase, are upregulated in PDAC primary tumors and liver metastases. Our finding of high levels of RON in PDAC is consistent with previous reports that link aberrant RON expression to pancreatic cancer progression and metastasis (Camp et al., 2007; Thomas et al., 2007; Chakedis et al., 2016). Our analysis of a limited number of samples suggests that upregulation of matriptase occurs prior to that of MSP. Similar to what we found with MSP and matriptase, an increase of RON expression has been noted in the PanIN stage (Thomas et al., 2007). It would be of interest to expand these studies and characterize further the sequence of events that promote the activation of this signaling pathway and neoplastic transformation. Conceivably, matriptase and MSP, especially its active form, may be explored as a potential biomarker for early detection of PDAC.

**FIGURE 6:**
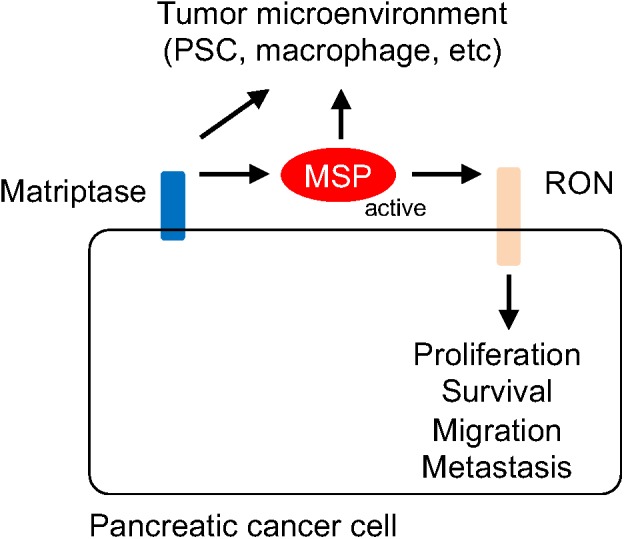
Schematic representation of the mechanism by which the matriptase-MSP-RON signaling axis modulates pancreatic cancer progression. Matriptase mediates maturation and activation of MSP, which in turn stimulates RON-modulated signaling events. It is conceivable that Matriptase and MSP may also contribute to remodeling of the tumor microenvironment, through regulating the functions of the stellate cell, the macrophage, or other immune cells.

The active form of MSP can bind RON, leading to its homo dimerization and activation of various downstream signaling events, such as RAS-MAPK and PI-3 Kinase pathways (Lu et al., 2007; Wagh et al., 2008). In addition, RON can modulate signaling events through forming heterodimers with other receptors such as c-MET (Follenzi et al., 2000; Benvenuti et al., 2011), EGFR (Peace et al., 2003; Thomas and Theodorescu, 2006; Liu et al., 2010), the platelet-derived growth factor receptor (PDGFR) (Kobayashi et al., 2009), and insulin-like growth factor 1 receptor (IGF1R) (Potratz et al., 2010; Jaquish et al., 2011). Activation of KRAS signaling has been linked to progression of pancreatic cancer. Indeed, KRAS mutations have been found in more than 90% human PanIN lesions as well as PDACs (Almoguera et al., 1988; Smit et al., 1988; Kanda et al., 2012; Murphy et al., 2013). Many KRAS coden 12 mutations, such as KRAS^G12D^, can lead to structural alterations that reduce the GTPase activity and prolong the GTP-bound, “active” form for signaling (Scheffzek et al., 1997). Notably, the signaling events upstream of KRAS, such as the one initiated from the RTK are required in transformation mediated by KRAS mutants (Huang et al., 2014). For example, EGFR is required for KRAS-induced neoplasm (Ardito et al., 2012; Navas et al., 2012). Thus, our findings suggest that the matriptase-MSP-RON signaling axis may represent an autocrine mechanism involved in promoting the transition from PanIN to PDAC by stimulating RTK and KRAS signaling (Figure 6).

Similar to what has been found in pancreatic cancer, activation of RON is also associated with tumorigenicity and metastasis of various other forms of human malignancy, such as bladder cancer, head and neck squamous cell carcinomas, breast cancer, ovarian cancer, and colorectal cancer (Chen et al., 2000; Zhou et al., 2003; Lin et al., 2004; Cheng et al., 2005; Lee et al., 2005). In particular, MSP has been shown to increase the invasive behavior and resistance to apoptosis in non-small cell lung cancer (Willett et al., 1998), as well as bone metastasis in breast cancer (Bhatt et al., 2007; Welm et al., 2007). A recent study indicate that MSP-RON can promote metastasis through activation of an MBD4-mediated DNA methylation program (Cunha et al., 2014). Inhibition of RON can enhance cancer cell sensitivity to chemotherapy drugs such as gemcitabine (Logan-Collins et al., 2010) or histone deacetylase inhibitors (Zou et al., 2013). The MSP-RON signaling network thus represents a potential therapeutic target for treatment of pancreatic cancer. However, it should be noted that because of the cross talk between RON and other RTKs, such as c-MET and erbB family members, a successful therapeutic approach may require simultaneous inhibition of multiple RTKs.

The MSP protein produced by pancreatic cancer cells may also be involved in cancer-stromal cell interaction through a paracrine mechanism. MSP was initially identified as a protein that modulates macrophage activities and RON has been shown to be involved in conversion to the M2-like macrophage (Skeel et al., 1991; Morrison et al., 2004; Sharda et al., 2011). Tumor-associated macrophages show characteristics of the M2-like phenotype and represent a major species of tumor-promoting immune cells in the tumor microenvironment (Grivennikov et al., 2010; Noy and Pollard, 2014). It would be of interest to determine whether matriptase and the activated forms of MSP proteins produced by cancer cells can modify macrophage behaviors. Alternatively, matriptase and MSP may contribute to shaping an immune suppressive landscape by acting upon other immune cells (Figure 6).

PDAC is characterized by a fibrotic and inflammatory microenvironment that is dominated by activated stellate cells. Our results show high levels of RON expression in the PDAC-associated PSCs. PSCs are myofibroblast like cells that are normally quiescent but become activated in damaged pancreas or the neoplastic niche, and produce collagen, fibronectin and fibrosis promoting growth factors and cytokines (Apte et al., 2009a,b; Erkan et al., 2012). Both PDGFR and EGFR are expressed in PSC and involved in its proliferation and functions (Apte et al., 1999; Blaine et al., 2009). It is conceivable that MSP can modulate cancer-stellate cell interaction through activation of RON and the interplay with other RTK signaling events in PSC (Figure 6).

In summary, our study demonstrates that the expression levels of matriptase, MSP and RON are significantly upregulated during the transition from the preneoplastic PanIN stage to the more advanced PDAC and metastatic lesions. These findings support the notion that activation of the matriptase-MSP-RON signaling network may play a critical role in neoplastic transformation in the pancreas.

## Materials and Methods

### Antibodies

The antibodies used in this study include: anti-MSP/MST1 (ab124787, Abcam, Cambridge, MA; MABF210, EMD Millipore, Burlington, MA, United States); anti-RON/MST1R (C-20, sc-322, Santa Cruz Biotechnology, Santa Cruz, CA, United States; MAB691, R&D Systems, Minneapolis, MN, United States); anti-alpha smooth muscle actin (α-SMA) (A2547, Sigma, St. Louis, MO, United States); anti-matriptase antibodies (#IM1014, EMD Millipore; D-7, sc-365482, Santa Cruz Biotechnology); HRP-conjugated secondary antibodies against mouse or rabbit were from Dako (Carpinteria, CA, United States).

### Human Tissues

Formalin-fixed and paraffin-embedded (FFPE) human specimens were obtained from the Cedars-Sinai Pathology archive and Biorepository with protocols approved by the Internal Review Board at the Cedars-Sinai Medical Center (IRB protocols #4201, #28197, and #34086). The tissue microarrays were obtained from US Biomax (Rockville, MD, United States).

### Transgenic Mice

All animal procedures were approved by the Cedars-Sinai Institutional Animal Care and Use Committee (IACUC) and performed in accordance with relevant guidelines and regulations (protocols #3935 and #8001). The KC and KPC mice derived from the LSL-KRAS^G12D/+^, LSL-p53^R172H/+^, and Pdx1-Cre (KPC) mice were described previously (Hingorani et al., 2003, 2005). The tissues were collected from 6-month-old mice, fixed in formalin and embedded in paraffin.

### Immunohistochemistry

Specimens of normal pancreas, PDAC, tissues with characteristics of PanINs, or pancreatic cancer metastasis to the liver, were analyzed. IHC was performed using a previously established protocol (Morvaridi et al., 2015). Briefly, the FFPE specimens were de-paraffinized, rehydrated, and subjected to heat induced antigen retrieval. After incubating in animal-free blocker for 30 min (SP-5030, Vector Laboratories, Burlingame, CA, United States), the samples were treated with primary antibody diluted at 1:200 to 1:1000 for 2 h or overnight. The sections were then washed three times in PBS, followed by incubation with secondary antibody for 1–2 h. The samples were washed, and specific stains were developed with the DAB Peroxidase substrate kit (SK4100, Vector Laboratories). The slides were mounted and scanned using Aperio Scanscope^®^AT Turbo (Leica Microsystems, Buffalo Grove, IL, United States). The results were evaluated using a binary scoring system, defined as either undetectable (negative) versus detectable (positive), or low versus high levels of staining. The correlation between gene expression and PDAC stages was examined using chi-squared test (GraphPad Prism, San Diego, CA, United States).

## Ethics Statement

This study was carried out in accordance with the recommendations of Cedars-Sinai Medical Center Internal Review Board (IRB) with written informed consent from all subjects. All subjects gave written informed consent in accordance with the Declaration of Helsinki. The protocol was approved by the Cedars-Sinai Medical Center Internal Review Board. This study was carried out in accordance with the recommendations of the Cedars-Sinai Institutional Animal Care and Use Committee (IACUC). The protocol was approved by the Cedars-Sinai Institutional Animal Care and Use Committee (IACUC).

## Author Contributions

QW conceived the study, designed and performed the experiments, analyzed and interpreted the data, created the figures, and drafted the manuscript. CL designed and performed the experiments, analyzed and interpreted the data, created the figures, and drafted the manuscript. SM, GL, CC, YK, and MK performed the experiments, analyzed and interpreted the data, and revised the manuscript and figures. ME, XY, NN, and SP analyzed and interpreted the data, and revised the manuscript.

## Conflict of Interest Statement

The authors declare that the research was conducted in the absence of any commercial or financial relationships that could be construed as a potential conflict of interest.
